# Root-associated bacterial and fungal communities of the endangered páramo bromeliad *Puya goudotiana*

**DOI:** 10.1371/journal.pone.0357104

**Published:** 2026-08-31

**Authors:** Nataly Rodríguez-Lugo, Luz H. Patiño, Tatiana M. Cáceres, Laura Vega, Stijn Hantson, Juan David Ramírez, Adriana Sanchez

**Affiliations:** 1 Departamento de Biología, Escuela de Ciencias e Ingeniería, Universidad del Rosario, Bogotá, Colombia; 2 Centro de Investigaciones en Microbiología y Biotecnología-UR (CIMBIUR), Escuela de Ciencias e Ingeniería, Universidad del Rosario, Bogotá, Colombia; 3 Programa en Ciencias del Sistema Tierra, Escuela de Ciencias e Ingeniería, Universidad del Rosario, Bogotá, Colombia; 4 Center for Global Health and Interdisciplinary Research, USF Genomics Program, Department of Global, Environmental and Genomic Health Sciences, College of Public Health, University of South Florida, Tampa, Florida, United States of America; University of Salento: Universita del Salento, ITALY

## Abstract

Páramo, a tropical high-altitude ecosystem, is threatened by climate change and land-use change. This ecosystem hosts unique biodiversity like the endangered bromeliad *Puya goudotiana*. While root-associated microbiomes are essential for plant survival and stress tolerance, the microbial communities associated with this species remain uncharacterized. Oxford Nanopore amplicon sequencing was used on root endosphere and bulk soil samples of *P. goudotiana*, targeting the 16S rRNA gene to evaluate bacterial communities, and the 18S rRNA gene as an exploratory marker for fungal communities. We assessed the taxonomic composition, diversity, functional profiles and co-occurrence networks. Microbial communities were highly differentiated by sample type, with roots exhibiting lower alpha diversity than bulk soil. The root microbiome showed higher prevalence of acidophilic taxa such as *Granulicella* and *Acidipila* and symbionts like *Bradyrhizobium*, whereas bulk soils were dominated by typical páramo taxa, including *Candidatus Solibacter*, *Candidatus Koribacter* and *Bryobacter*. Both bulk soil and roots were characterized by a high abundance of saprotrophic fungi (e.g., *Psilocybe*) and potential pathogens such as *Fusarium*, *Botrytis* and *Puccinia*. Functional predictions indicated higher prevalence of chemoheterotrophic functions in the roots, while nitrogen and sulfur cycling functions were enriched in bulk soil. Notably, and contrary to prior expectations, co-occurrence networks were more complex in the root endosphere than in bulk soil, suggesting that rhizosphere filtering promotes structured microbial assemblages despite reducing overall diversity. These findings provide a first microbial baseline for *P. goudotiana* and open new perspectives for understanding plant–microbe interactions and enhancing páramo vegetation resilience under climate change.

## Introduction

Páramos are tropical high-altitude Andean ecosystems, occurring between approximately 3,000–5,000 meters above sea level (masl). They provide key ecosystem services, including water regulation to urban centers, carbon sequestration and the maintenance of exceptional levels of biodiversity, which has led to their recognition as a global biodiversity hotspot [1,2]. Páramo ecosystems are characterized by extreme environmental conditions, such as high daily temperature fluctuations, intense UV radiation and strong precipitation variability [3]. Precipitation regimes vary considerably across páramos, ranging from monomodal to bimodal seasonal patterns. Annual precipitation spans from relatively dry systems with around 1,000 mm to wet ones exceeding 3,200 mm [4]. In most páramos, July and August are the wettest months, while January and February are the driest. Despite their ecological importance, páramos are increasingly threatened by climate change and land-use changes, such as agricultural expansion and livestock grazing, which pose substantial risks to endemic plants species [5,6].

Soils are highly diverse and complex environments. They harbor vast microbial communities, including bacteria, fungi, protozoa, etc. The composition and function of these communities are shaped by soil properties, environmental conditions and biotic interactions [7,8]. Within this matrix, plant roots primarily influence the immediately surrounding soil. This zone is known as the rhizosphere. Root shape it through root exudation, the secretion of carbon-rich compounds into the soil [9]. This process drives rhizosphere filtering, whereby roots selectively recruit a subset of the bulk soil microbial community [10]. Filtering produces a hierarchically structured community. The endosphere represents a further-filtered subset, assembled from the rhizosphere microbiota itself [11]. As a result, root-associated communities often differ taxonomically and functionally from those in bulk soil, and their microbial networks are frequently less complex, potentially reflecting differences in resource availability and environmental filtering within the rhizosphere [12].

These root-associated communities influence plant performance by contributing to nutrient acquisition (e.g., nitrogen, phosphorus and sulfur), pathogen suppression and plant responses to abiotic and biotic stressors [10,13,14]. Among soil microorganisms, bacteria and fungi account for the largest proportion of the microbial biomass and play key roles in plant health and survival [8]. For instance, beneficial bacteria such as those belonging to the genus *Pseudomonas,* produce antimicrobial compounds and modulate phytohormone signaling [15,16]. Beneficial fungi like arbuscular mycorrhizae, colonize root cortical cells, facilitating nutrient and water uptake [17].

Characterizing microbial communities assembly of páramo plants is an important first step toward understanding how microorganisms are associated with plant persistence in high-elevation ecosystems. Microbial associates can enhance plant performance under extreme conditions by mediating tolerance to cold stress, phytohormone production and synthesizing antibiotic compounds [18]. This knowledge is particularly relevant under ongoing climate change, as páramo vegetation is expected to experience upward range shifts, habitat contraction and increased risk of local extinction [19]. Integrating microbiome research into páramo ecology may therefore improve understanding of threatened plant species and their associated belowground communities, while also informing future conservation and restoration strategies under climate change.

Within the páramo, the Bromeliaceae family is among the most conspicuous plant groups, predominately represented by the genus *Puya* [20]. Species of *Puya* play key ecological and cultural roles by providing food resources for local communities [21], fauna such as the Andean bears (*Tremarctos ornatus*) [22] and nectar for hummingbirds [23]. However, *Puya* is also considered a highly threatened genus [24,25]. In particular, *Puya goudotiana*, a species endemic to the Colombian Eastern Cordillera, has recently been reassessed from Least Concern to Endangered [24].

Despite the ecological and conservation relevance of *Puya*, research on belowground microbial dynamics in páramo ecosystems has focused predominantly on other dominant rosette-forming plants, particularly species of *Espeletia*. Previous studies on *Espeletia* have identified root-associated microbial communities dominated by Acidobacteria, Actinobacteria and Proteobacteria, including key taxa like *Candidatus Solibacter* and *Candidatus Koribacter* [26]. These communities also exhibit a high abundance of filamentous fungi and yeasts, as well as predicted functional enrichment in cellulose degradation [27]. In contrast, the microbial communities associated with *Puya* remain largely unexplored [28,29], representing a critical knowledge gap in our understanding of plant-microbe interactions in these high-Andean ecosystems.

Therefore, this study aims to characterize the root-associated bacterial and fungal communities (herein microbial communities) of *Puya goudotiana.* We used Oxford Nanopore amplicon sequencing of the 16S rRNA gene to target bacterial communities and the 18S rRNA gene to explore fungal communities. For each compartment, roots and bulk soil, we evaluated taxonomic composition, diversity, predicted functional profiles and co-occurrence network structure. We hypothesize that (i) root-associated communities would exhibit lower microbial diversity than bulk soil communities as a consequence of rhizosphere filtering processes; (ii) root-associated communities would exhibit enrichment of predicted functions related to nutrient cycling and organic matter utilization; and (iii) microbial co-occurrence networks in roots would be less complex than those in bulk soil.

## Materials and methods

### Study site and sample collection

Samples for this study were collected in July of 2024, in the páramo of Matarredonda Natural Reserve, Choachí municipality, Colombia (S1 Fig). This páramo is located in the Cruz Verde-Sumapaz páramo complex at an elevation of ca. 3,300 masl., has a mean annual temperature of 8.8 ºC and precipitation ranges between 1,000 and 1,500 mm yr^-1^ [30,31]. This páramo is characterized by a long rainy season from April to November and a dry season between December and March. We selected sampling locations in the páramo based on a previous study [2]. This research was conducted under the ANLA permit 001431 of July 2024.

Within this area, twelve similarly sized adult individuals of *Puya goudotiana*, with rosettes ~ 50 cm wide and ~ 60 cm tall, were randomly selected. Under aseptic conditions, we collected fine root samples (10–25 cm depth) and their corresponding surrounding bulk soil, storing each in 50 mL collection tubes (n = 12 per sample type). To ensure that root samples could be attributed to the target individual rather than to neighboring plants, a minimum inter-individual distance of 2 m was maintained during sampling. Additionally, roots were manually traced from the collection point back to the base of the target plant prior to harvesting, confirming their identity before excision. This combination of spatial spacing and individual-level root tracking minimized the risk of inadvertently sampling roots from adjacent individuals. Samples were transported on the same day in a portable cooler at 4ºC to the laboratory of the Grupo de Investigaciones Microbiológicas-UR (GIMUR) for storage at –30 ºC.

To account for potential variations caused by fire activity in this páramo [2], soil physicochemical properties were analyzed. We collected composite soil cores (approximately 1000 g, at depths up to 15 cm) distributed across the sampling area. Soils were air-dried for a week and then sieved. The physicochemical analysis was conducted by the Instituto Geográfico Agustín Codazzi (Bogotá, Colombia), which evaluated pH, percentage of organic matter, cationic relations, soil texture and cation exchange capacity (See S1 Table for analytical protocols).

### DNA extraction and PCR

We followed the root sample preparation protocol described by [32], which involves sequential removal of loosely adhering bulk soil and rhizosphere soil by vortexing and sonication, retaining the washed root fraction that represents the root endosphere community. Bulk soil DNA extraction was done using the DNeasy PowerSoil Pro Kit (catalog number 47014; Qiagen) and for the roots we used the NucleoSpin Soil Kit (catalog number 740780.50; MACHEREY-NAGEL) following the manufacturer’s recommendations. To account for potential contaminants during DNA extraction, we assessed the DNA quality according to the 260/280 and 260/230 ratios using a Nanodrop One Spectrophotometer (catalog number 13400518; Thermo Scientific). The use of different extraction kits for roots and bulk soils was determined by preliminary extraction tests to ensure optimal DNA quality, preventing potential failures during downstream PCR and sequencing.

For bacteria, we amplified the full length 16S rRNA gene using the 27F 5’-AGAGTTTGATCCTGGCTCAG-3’ and 1492R 5’-GGTTACCTTGTTACGACTT- 3’ primers [33]. For fungal community profiling, amplification of the ITS region was first attempted but consistently failed to produce reliable amplicons across all samples, likely due to the high concentration of host DNA potentially causing PCR inhibition. We therefore employed the V4-V5 region of 18S rRNA gene as an exploratory alternative, using the 566F 5’-CAGCAGCCGCGGTAATTCC-3’ and 1289R 5’-ACTAAGAACGGCCATGCACC-3’ primers [34], which have been shown to amplify reliably across diverse environmental samples. We acknowledge that 18S provides lower taxonomic resolution for fungi than ITS, particularly at the species and genus level, and that our fungal community profiles should therefore be interpreted as an exploratory characterization of broad taxonomic patterns rather than a definitive community census. Both PCR mixtures contained 6.25 µL of LongAmp Taq 2X Master Mix (catalogue number M0287L; New England Biolabs), 2.25 µL of molecular-grade water, 1 µM of each primer and 1.25 µL of root or bulk soil DNA.

PCR thermal profile for 16S rRNA gene amplification consisted of: initial denaturation at 94 ºC for 30 seconds, followed by 30 cycles of 94 ºC for 30 seconds, 47.9 ºC for one minute and 65 ºC for 1 minute and 15 seconds, and final extension at 65 ºC for 10 minutes. Whereas for the amplification of the V4-V5 region of the 18S rRNA gene, the thermal profile used was: initial denaturation at 94 ºC for 30 seconds, followed by 30 cycles of 94 ºC for 30 seconds, 54.6 ºC for one minute and 65 ºC for 30 seconds, and final extension at 65 ºC for 10 minutes. The amplicon bands (approximately 1500 bp and 700 bp for 16S rRNA and 18S rRNA, respectively) were confirmed using a 2% agarose gel. A negative control was included during PCR preparation and gel electrophoresis to ensure the absence of contamination during amplification. DNA concentration of amplicons was evaluated using the Qubit™ 3 Fluorometer (catalogue number Q33216; Invitrogen), and all samples were standardized to a final concentration of 11 ng/µL.

### Amplicon sequencing and bioinformatic analysis

We generated a sequencing library for the PCR products using the Ligation Sequencing Kit (catalogue number SQK-LSK109; Oxford Nanopore Technologies) following the manufacturer’s instructions. Two negative controls were also included during library preparation to account for sequencing quality. We then loaded the products and controls into an R10.4.1 flow cell (FLO-MIN114) and sequenced using the MinIonMk1C instrument with MinKNOW software version 22.10.7.

Basecalling and demultiplexing were performed using dorado (https://github.com/nanoporetech/dorado). For the quality assessment of the FASTQ files, we used the NanoPack tools [35], to visualize the reads summary statistics (NanoPlot) and filter them (NanoFilt) with a minimum mean quality score of 15 and lengths between 100–1700 bp and 100–900 bp for 16S rRNA and 18S rRNA, respectively. Taxonomic assignment of filtered reads was done with Kraken 2 [36]. We used the SILVA v.138.1 [37] database for 16S reads. For fungal 18S reads, we used the PlusPF database (build date 12/28/2024) (https://benlangmead.github.io/aws-indexes/k2), which incorporates eukaryotic sequences and provided adequate coverage for broad-level fungal classification in this exploratory context. We note that while ITS-based databases such as UNITE offer superior resolution for fungal taxonomy, the use of 18S and the PlusPF database was a pragmatic decision given the ITS amplification failure described above, and results should be interpreted accordingly. These results were visualized with Pavian [38]. All taxa corresponding to mitochondria and non-fungal eukaryotes were removed. Additionally, taxonomic profiling of the sequenced negative controls confirmed a negligible background signal (< 11 reads for bacterial or fungal genera).

### Data analysis

All the statistical analyses were conducted in R (v.4.5.0) [39] with the phyloseq package v.1.52.0 [40] at the genus level. Differentially abundant taxa between sample types were identified using ANCOM-BC2 v.2.10.0 [41]. A prevalence cutoff of 10% and library cutoff of 1,000 reads were implemented. Structural zeros were identified and excluded from the analysis. Results were considered significant at a Holm-corrected *p* value < 0.05. To assess fungal and bacterial diversity, alpha diversity measures were calculated from taxonomic assignments, including the abundance-based coverage estimator (ACE) to estimate richness and the Shannon and Simpson indexes to characterize both microbial communities. Statistical comparisons between groups were performed with Wilcoxon rank-sum tests. Principal Coordinates Analysis (PCoA) based on Bray–Curtis dissimilarities were performed to visualize overall patterns in bacterial and fungal community composition. Differences in microbial communities between sample types were evaluated using permutational multivariate analysis of variance (PERMANOVA) with plant individual as a blocking factor, using the vegan package v.2.6–10 [42]. We also conducted a permutational analysis of multivariate dispersion (PERMDISP) across sample types. During ordination analysis, one root endosphere sample (number six) consistently clustered apart from the remaining samples in the bacterial and fungal datasets and was therefore considered a potential outlier, likely due to technical variation. To evaluate its influence on the results, beta diversity analyses were performed both including and excluding this sample. However, the overall clustering patterns and statistical significance remained unchanged. Therefore, the sample was excluded from the final PCoA visualizations to improve interpretability, resulting in an effective sample size of n = 11 for the root compartment. PCoA plots comparing the distribution of bacterial and fungal communities with and without the outlier are provided in S2 Fig.

Functional prediction analysis of the bacterial genera was performed with FAPROTAX [43]. Additionally, to assess the ecological role of the fungal genera, trophic mode was assigned using the FungalTraits database (v.1.2) [44], based on the primary lifestyle. We implemented the category “Other” to the genera whose primary lifestyles were defined as endophytic or associated with animals [45]. Comparisons of predicted bacterial functions, fungal trophic modes and fungal lifestyles between sample types were conducted with Wilcoxon tests on relative abundances with FDR corrected *p* values.

Finally, co-occurrence networks of bacterial, fungal and cross-domain communities for root and bulk soil samples were calculated using the microeco v.1.16.0 [46] and meconetcomp v.0.6.1 [47] R packages. To ensure network robustness, only genera with a relative abundance higher than 0.02% and a prevalence ≥ 50% were retained for analysis [48]. Strong Spearman’s correlations (|ρ| > 0.60) with FDR corrected *p* values < 0.01 were selected for network construction. Subsequently, networks were exported from RStudio with the rgexf package v.0.16.3 [49] and then visualized in Gephi v.0.10.1 [50] using the Fruchterman–Reingold algorithm [51].

## Results

### Soil physicochemical properties

Soil physicochemical properties exhibited low to moderate variability across the study area (S2 Table). While pH, total nitrogen (TN) and oxidable organic carbon (OOC) remained relatively homogeneous, greater heterogeneity appeared in nutrient levels and soil texture, likely reflecting local gradients. This limited edaphic variability suggests that the variations in microbial communities described below are driven by the plant-soil interface rather than by soil properties or past disturbances.

### Taxonomic composition and diversity of bacterial and fungal communities

Following the sequencing of the 16S rRNA gene, we obtained a total of 537,206 reads successfully assigned to the genus level. Taxonomic classification identified 1,630 bacterial genera. After removing taxa with zero read counts, 1,525 genera were retained for downstream analyses. Of these, 230 and 480 were unique to roots and bulk soil, respectively, and 815 were shared between compartments. At the family level, the taxonomic profile revealed distinct dominance patterns between niches. The root communities were primarily dominated by Acidobacteriaceae (Subgroup 1) (13.40%), Xanthobacteraceae (13.10%) and Beijerinckiaceae (8.00%), whereas the bulk soil was characterized by a high prevalence of uncultured Acidobacteriales (11.90%), Gemmataceae (9.29%) and Solibacteraceae (Subgroup 3) (8.63%) (S3 Table).

**Fig 1:**
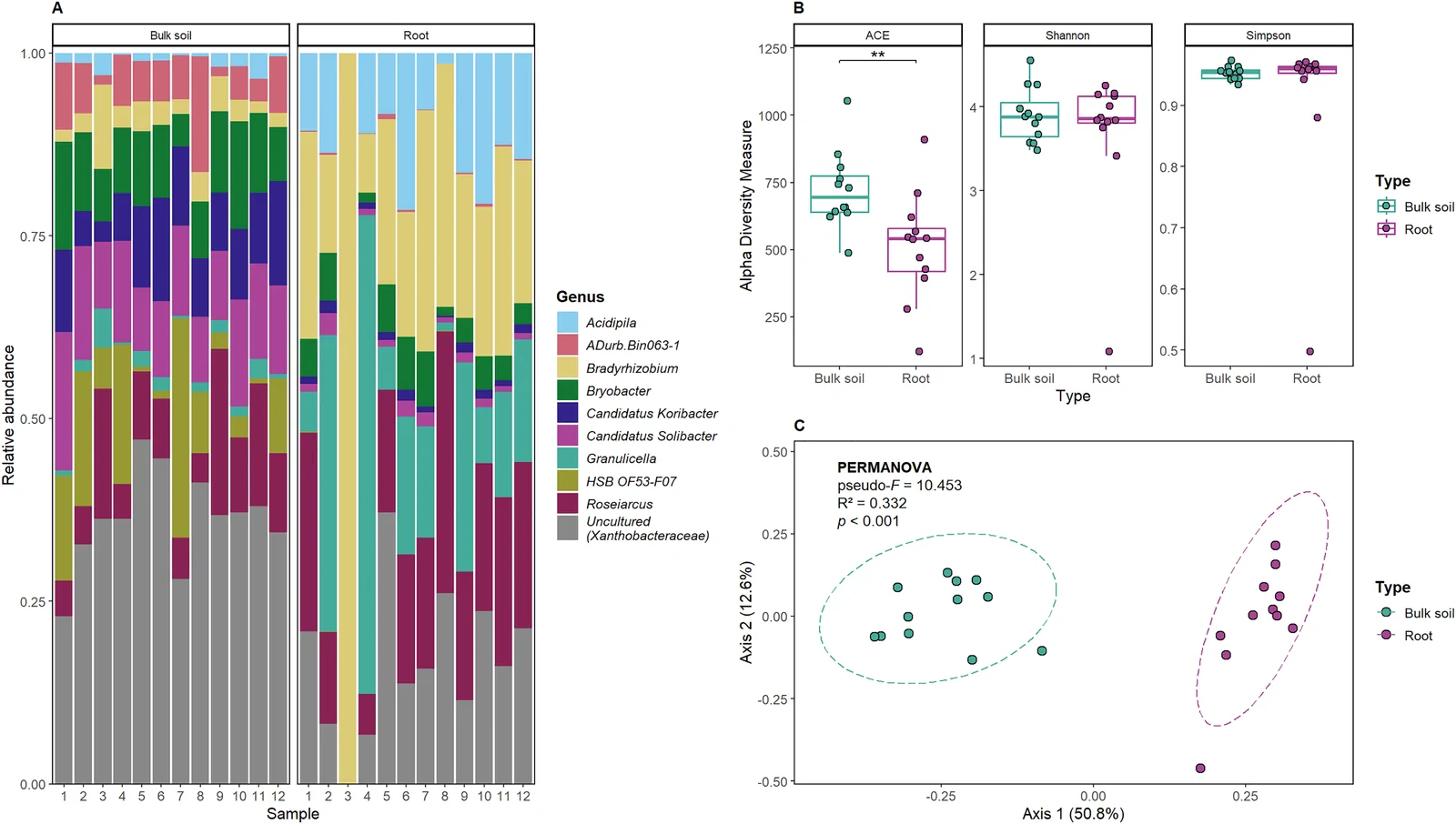
Diversity and taxonomic composition of bacterial communities. (A) Relative abundance profiles of the ten most dominant bacterial genera in root and bulk soil samples. Note that taxa outside the top ten are not included for visualization clarity. (B) Alpha diversity measures of bacterial communities at the genus level. Asterisks indicate significant differences between sample types according to Wilcoxon rank-sum tests: *, *p* < 0.05; **, *p* < 0.01; ***, *p* < 0.001. (C) Principal Coordinates Analysis (PCoA) based on Bray–Curtis dissimilarities of bacterial communities at the genus level. PERMANOVA results are shown in the plot.

At the genus level, bacterial communities were dominated by an uncultured genus of the Gemmataceae family, *Candidatus Solibacter*, an uncultured genus of the Xanthobacteraceae family, *Roseiarcus* and HSB OF53-F07 in the bulk soil, accounting for 12.30, 6.38, 5.91, 5.20 and 5.20% of the total relative abundances, respectively (Fig 1A). In contrast, *Granulicella* (7.81%)*, Bradyrhizobium* (7.32%)*, Roseiarcus* (6.49%)*, Acinetobacter* (5.54%) and an uncultured genus of the Xanthobacteraceae family (4.74%) were dominant in the roots (Fig 1A) (S4 Table). Additionally, we identified a total of 42 differentially abundant genera; among these, three (*Microbacterium, Jatrophihabitans* and *Rudaea*) were significantly enriched in the roots, while 39 were enriched in the bulk soil (ANCOM-BC2, *p* < 0.05) (S3 Fig).

Regarding bacterial alpha diversity, bulk soil communities exhibited significantly higher richness than root communities. However, Shannon and Simpson indices remained consistent across sample types (Fig 1B). Moreover, beta diversity analysis revealed a significant differentiation in community composition between root and bulk soil samples (Fig 1C). While the multivariate dispersion analysis was also significant between sample types (PERMDISP, *F*: 5.295, *p*: 0.028), the clear clustering pattern in the PCoA suggests a compositional separation between compartments.

Fungal community profiling based on the 18S rRNA gene sequencing revealed that the fungal reads represented 18.87 ± 12.93% of the total eukaryotic reads per sample. Within this fraction, 115,691 reads were taxonomically resolved at the genus level, classified into 63 fungal genera. Of these, 59 were shared between bulk soil and root samples, whereas only 4 were unique to bulk soil. This is markedly a lower richness estimate than the bacterial dataset, consistent with the known lower discriminatory power of 18S relative to ITS for fungal community characterization. Results should therefore be interpreted as indicative of broad community patterns rather than a complete census of fungal diversity. At the family level, the fungal composition underwent a notable transition from soil to roots. While the bulk soil was characterized by a relatively similar abundance of Podosporaceae (11.50%), Nectriaceae (10.40%) and Strophariaceae (8.71%), the root-associated communities became heavily dominated by Strophariaceae, which accounted for one-third of the total abundance (33.10%), followed by Nectriaceae (11.30%) and Ceratobasidiaceae (8.18%) (S5 Table).

**Fig 2:**
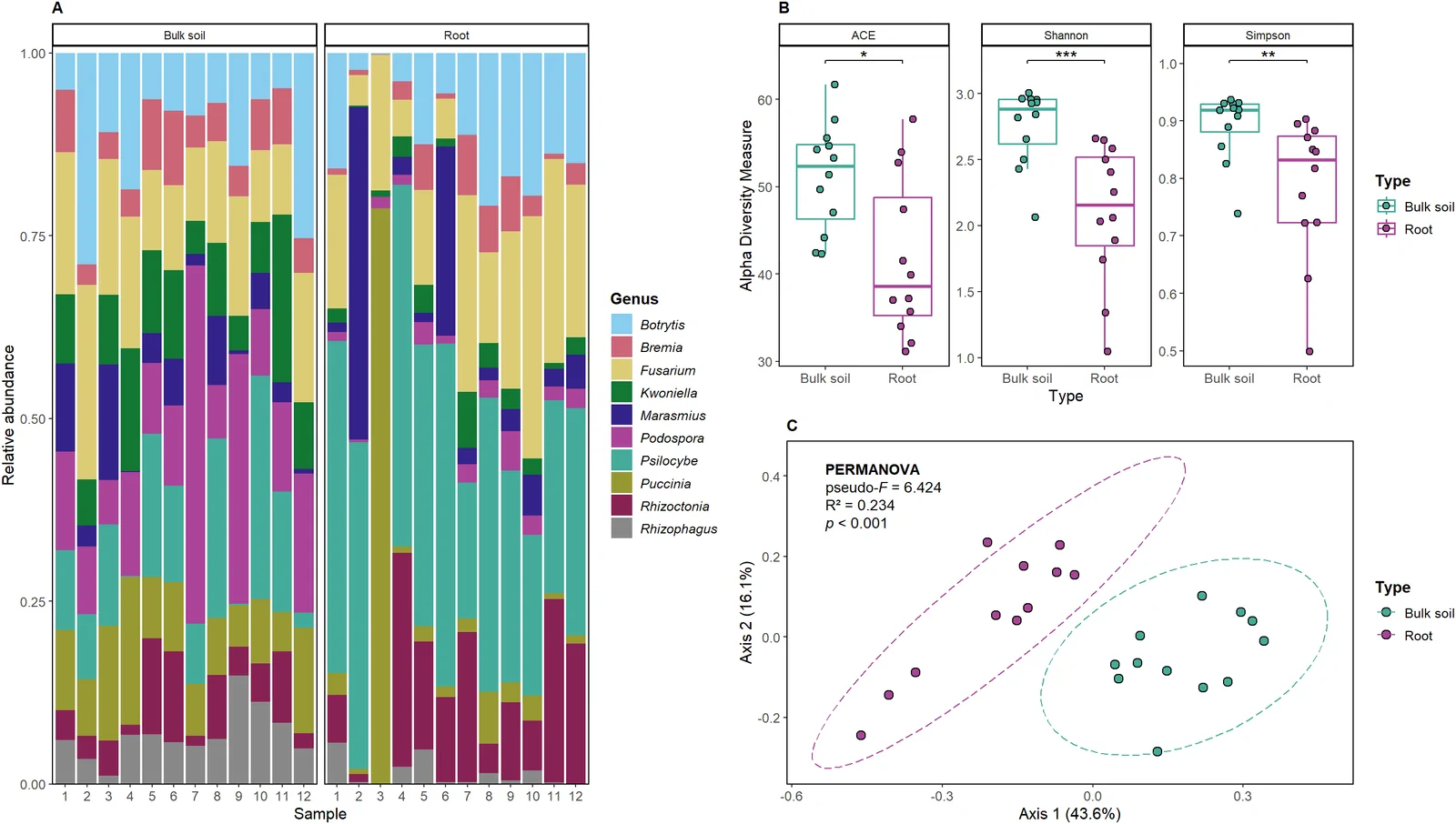
Diversity and composition of fungal communities. (A) Relative abundance profiles of the ten most dominant fungal genera in root and bulk soil samples. Note that taxa outside the top ten are not included for visualization clarity. (B) Alpha diversity measures of fungal communities at the genus level. Asterisks indicate significant differences between sample types according to Wilcoxon rank-sum tests: *, *p* < 0.05; **, *p* < 0.01; ***, *p* < 0.001. (C) Principal Coordinates Analysis (PCoA) based on Bray–Curtis dissimilarities of fungal communities at the genus level. PERMANOVA results are shown in the plot.

When examining the communities at the genus level, *Podospora, Fusarium, Psilocybe, Botrytis* and *Puccinia* were dominant in the bulk soil, representing 11.80, 10.80, 8.77, 8.49 and 7.02% of the relative abundances, respectively (Fig 2A). Whereas in the root endosphere, *Psilocybe* (27.20%)*, Fusarium* (13.70%)*, Rhizoctonia* (10.20)*, Botrytis* (8.93%) and *Puccinia* (7.30%) were the most abundant genera (Fig 2A and S6 Table). No differentially abundant fungal genera were identified between root endosphere and bulk soil samples by ANCOM-BC2. This result should be interpreted cautiously in light of the small number of genera recovered (n = 63) and the modest sample size (n = 12 per compartment), both of which substantially reduce statistical power for differential abundance detection.

Fungal alpha diversity was significantly higher in bulk soil communities than in root endosphere communities (Fig 2B). Also, the PCoA of beta diversity revealed distinct and significant clustering by sample type, supported by PERMANOVA results (Fig 2C). Although the homogeneity of multivariate dispersions also differed significantly between sample types (PERMDISP, *F*: 5.34, *p*: 0.032), the clear separation of groups along Axis 1 suggests substantial compositional turnover between compartments. Together, these results suggest that the root endosphere exerts a degree of filtering on fungal assemblages, though the mechanistic basis of this pattern awaits confirmation by ITS-based approaches.

### Functional profiling of bacterial and fungal communities

**Fig 3:**
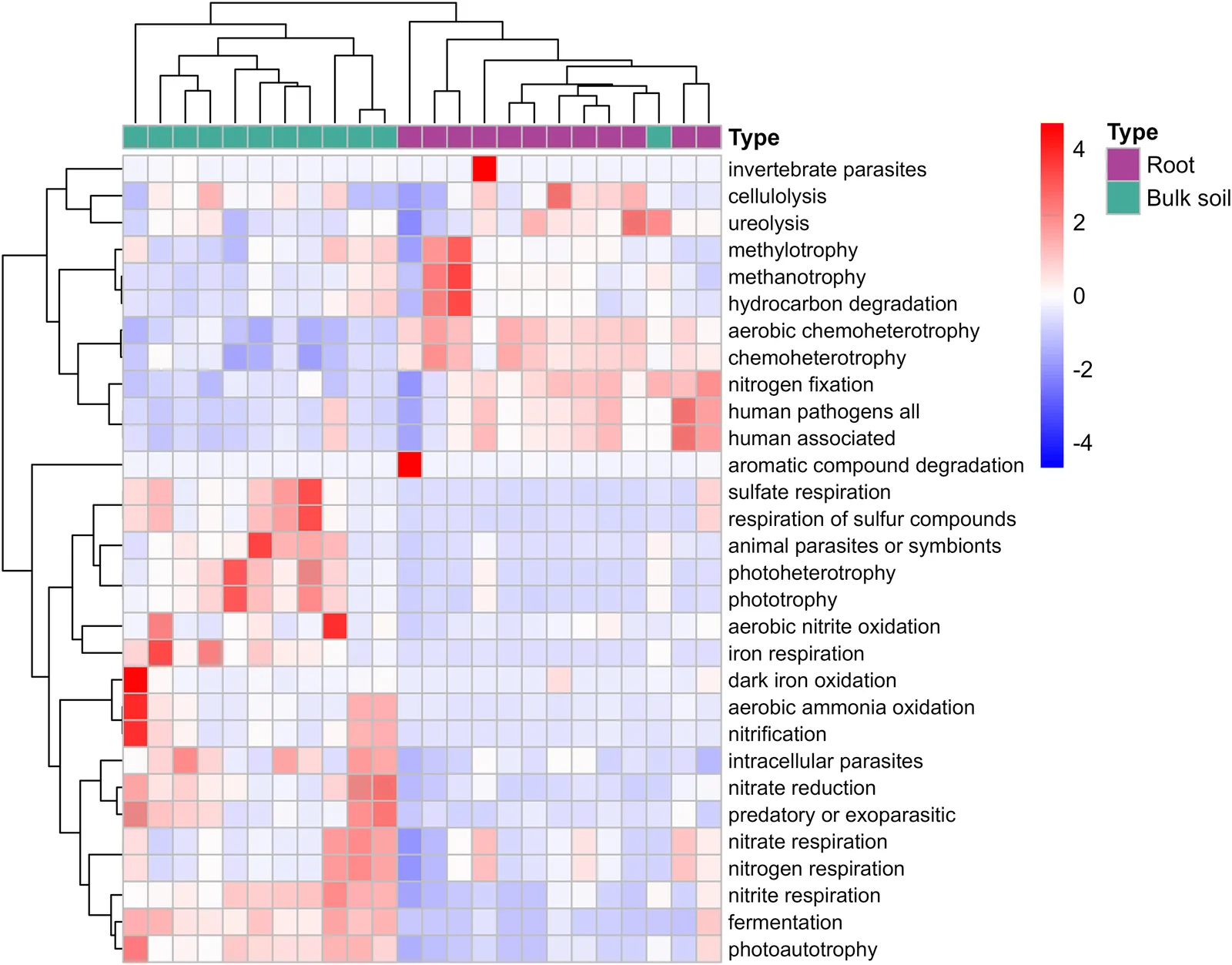
Heatmap of the top 30 predicted bacterial functions using the FAPROTAX database. Color intensity represents the *z*-score of the relative abundance of each function. Samples are grouped according to sample type.

Bacterial functions were predicted using FAPROTAX. Overall, 66 functional annotations were identified across all samples. There was a clear differentiation of functions between sample types (Fig 3), with Wilcoxon rank-sum tests confirming significant shifts in relative abundance for 30 categories (S7 Table). Root-associated communities showed higher relative abundance of taxa associated with predicted chemoheterotrophic functions and nitrogen fixation. In contrast, the bulk soil displayed a distinct functional signature, characterized by a significant overrepresentation of N-cycle processes (such as nitrite respiration, nitrification and nitrate respiration), S-cycle pathways (e.g., sulfate respiration), fermentation, iron respiration and phototrophy.

**Fig 4:**
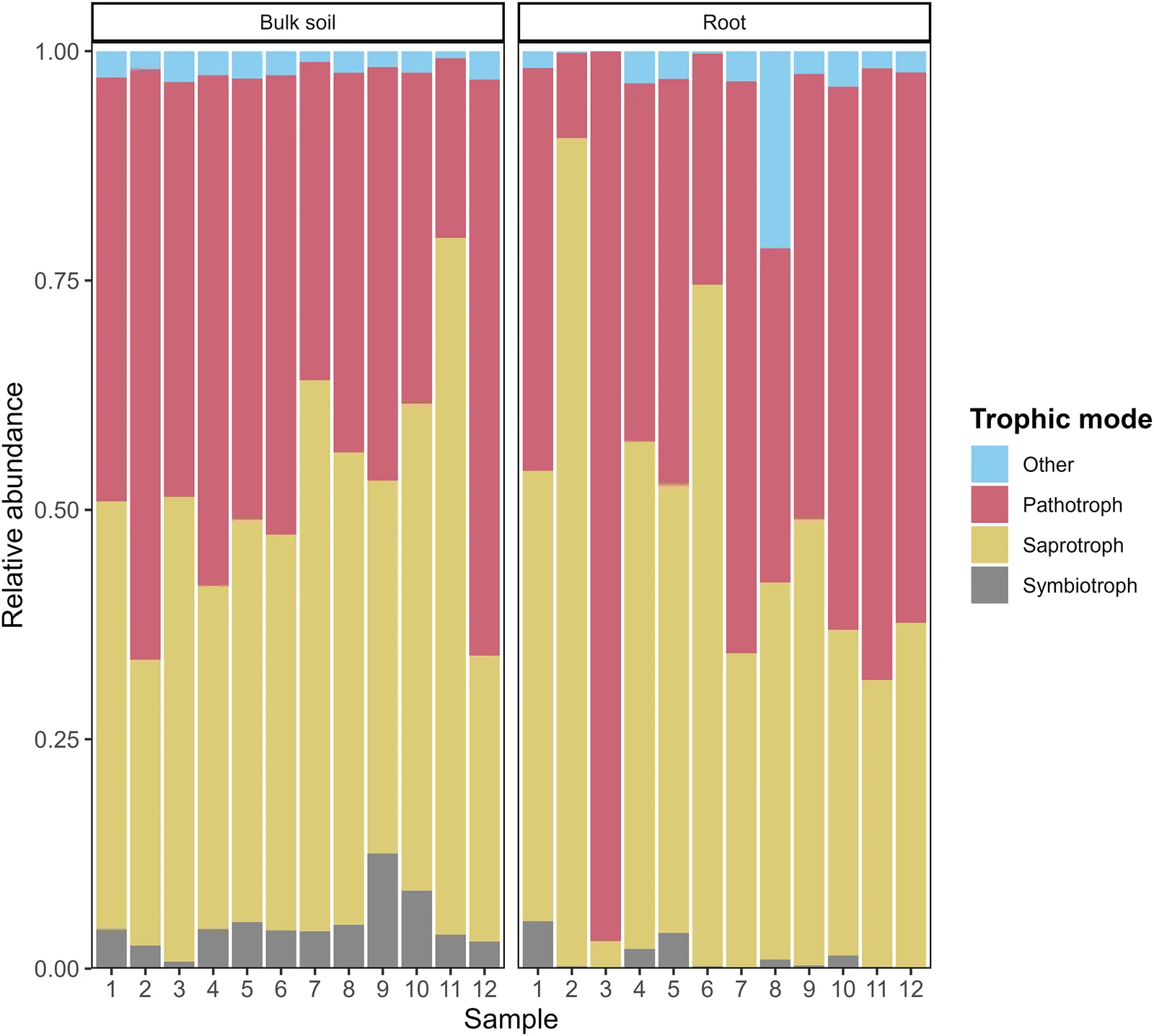
Relative abundance profiles of fungal trophic modes in root and bulk soil samples.

On the other hand, we used FungalTraits to identify the trophic modes of the fungal communities. The most prevalent categories were pathotrophs and saprotrophs, which together accounted for over 92% of the total relative abundance across all samples (Fig 4). In the bulk soil communities, saprotrophs and pathotrophs showed a balanced distribution, representing 47.10% and 45.70% of the community, respectively. In the root communities, the average abundance of pathotrophs increased slightly to 49.30% and in several samples, such as numbers 3, 7 and 11, they were significantly overrepresented, exceeding 60% of the community. Notably, symbiotrophs were consistently more restricted in both sample types, representing a minor fraction of the community (ranging from 1.2% in roots to 4.8% in bulk soil). This trophic mode was significantly more abundant in the bulk soil (S8 Table) and primarily composed of the arbuscular mycorrhizal genus *Rhizophagus.* Additionally, several types of saprotroph fungi were found more abundant in the bulk soil, whereas litter saprotrophs predominated in the root endosphere (S8 Table).

### Co-occurrence network analysis

**Fig 5:**
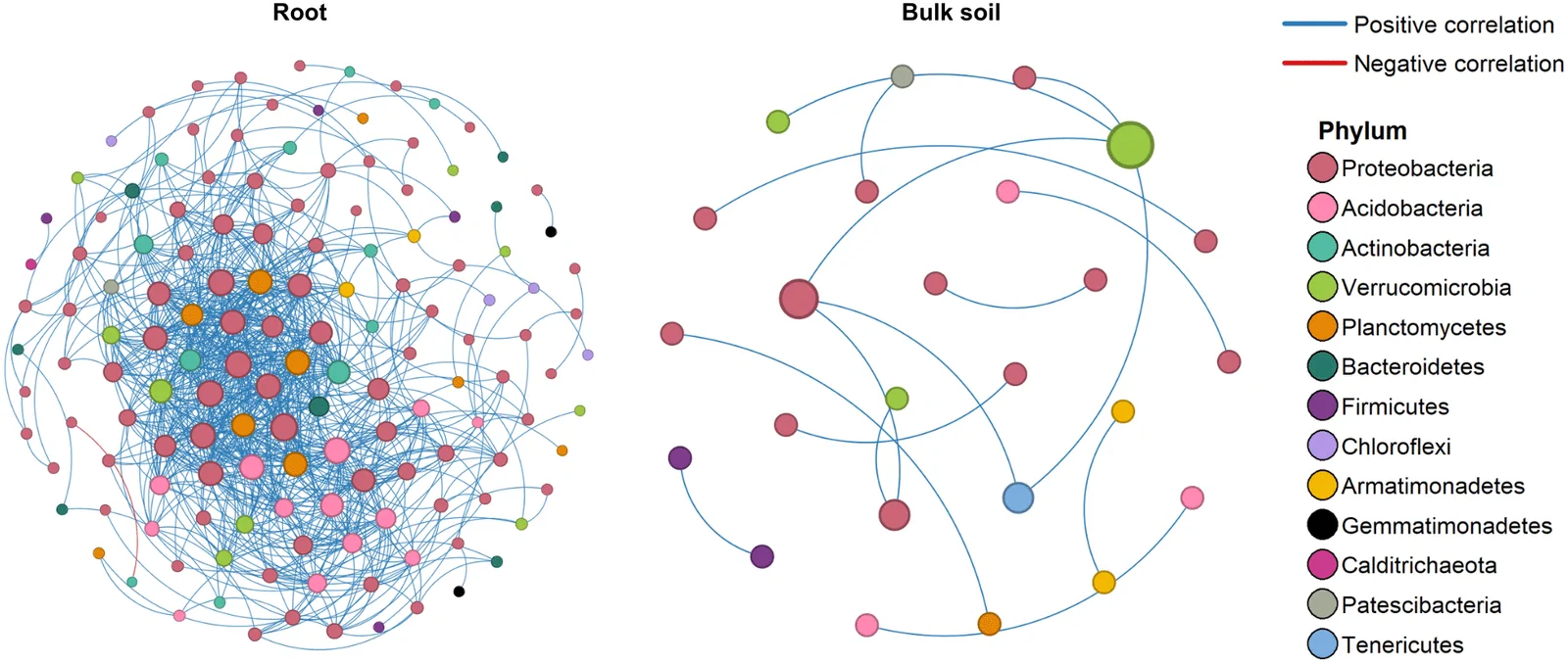
Bacterial co-occurrence networks of root and bulk soil samples. Node size is proportional to the degree number. Nodes are resolved at the genus level.

Bacterial communities showed positive and strong co-occurrence relationships in roots and bulk soils (Fig 5). The root network consisted of 99.88% positive and 0.12% negative correlation edges, whereas the bulk soil network was composed entirely of positive correlations. The most influential phylum in both networks was Proteobacteria, representing 55.94% of the connections in the roots and 48% in the bulk soil. Following Proteobacteria in the roots, Acidobacteria, Actinobacteria, Verrucomicrobia and Planctomycetes were the most prevalent phyla, accounting for 9.09%, 7.69%, 6.29% and 6.29% of the connections, respectively. Similarly, in the bulk soil, after Proteobacteria, most nodes belonged to the phyla Verrucomicrobia (12%), Acidobacteria (12%), Firmicutes (8%) and Armatimonadetes (8%). The bacterial root network consisted of 143 nodes and 842 edges, while the bulk soil network had 25 nodes and 16 edges. The two networks shared 20 nodes, while 123 were unique to the root network and 5 to the bulk soil network. According to various network topological metrics, such as average degree, density and clustering coefficient, the root network exhibited higher complexity compared to the bulk soil network (S9 Table).

Similar to the bacterial co-occurrence networks, the fungal root network showed a higher complexity than the fungal bulk soil network (S4 Fig and S9 Table). Additionally, only positive correlation edges were identified in both networks. In the root network, the most dominant phyla were Ascomycota and Basidiomycota, with 69.23% and 23.08% of the connections, respectively. Oomycota and Glomeromycota represented together 7.7% of the connections. In contrast, the fungal bulk soil network had the lowest node and edge count, with Basidiomycota and Ascomycota accounting for 66.67% and 33.33% of the nodes, respectively.

**Fig 6:**
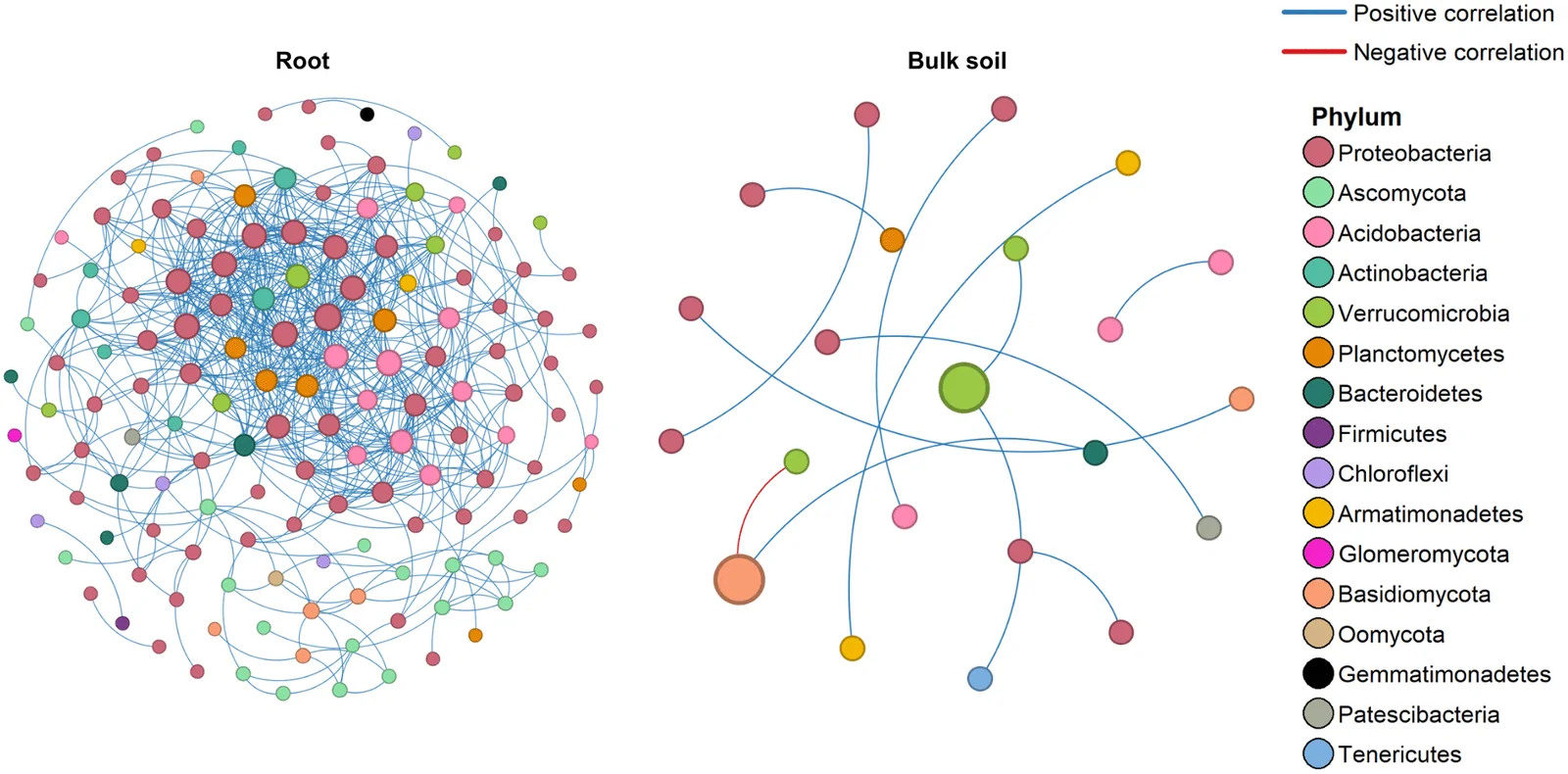
Cross-domain co-occurrence networks of root and bulk soil samples. Node size is proportional to the degree number. Nodes are resolved at the genus level.

Cross-domain network complexity did not differ from that of the fungal and bacterial networks (Fig 6 and S9 Table). The root network contained 143 nodes and 654 edges, all positively correlated. It was dominated by Proteobacteria (48.95% of connections), followed by Ascomycota (12.59%), Acidobacteria (9.09%), Actinobacteria (4.9%), and Verrucomicrobia (4.9%); Basidiomycota accounted for only 3.5%. The bulk soil network was much smaller, with 22 nodes and 12 edges, of which 91.67% were positive and 8.33% negative. Proteobacteria again dominated (36.36%), followed by Verrucomicrobia and Acidobacteria (13.64% each) and Armatimonadetes and Basidiomycota (9.09% each). Notably, Ascomycota was absent from this network. The two networks shared 17 nodes, with 126 nodes unique to the root network and 5 unique to the bulk soil network.

## Discussion

### Composition and diversity of fungal and bacterial communities

Consistent with our first hypothesis, microbial diversity was lower in the root endosphere than in bulk soil, particularly within fungal communities. Root-associated communities likely represent a subset of the surrounding bulk soil microbiota, a pattern widely attributed to rhizosphere and endosphere filtering processes that selectively reduce microbial richness and diversity near and within roots [13]. The enrichment of copiotroph bacteria, including members of the Burkholderiaceae and Beijerinckiaceae families, in the root endosphere is consistent with the higher availability of labile carbon in root-proximal environments, whereas oligotrophic taxa such as Pedospheraceae and Ktedonobacteraceae were more prevalent in bulk soils [52].

Within bacterial communities, oligotroph taxa commonly associated with acidic, Zn and Fe-rich soils characteristic of the páramo [53], including *Candidatus Solibacter, Candidatus Koribacter* and *Bryobacter*, were more abundant in bulk soil [54]. In contrast, root-associated communities were enriched in taxa frequently reported from acidic environments, including *Granulicella*, *Acidipila* and *Roseiarcus* [55–57], as well as *Bradyrhizobium,* a genus that has been reported as dominant in the root-associated communities of *Espeletia* spp. [58]. These patterns are consistent with host- and soil-mediated filtering of the bulk soil microbial pool, though the specific mechanisms driving taxon-level enrichment in *P. goudotiana* endosphere communities remain to be experimentally determined.

Regarding the fungal community characterization, the modest proportion of fungal reads per sample of the total eukaryotic reads requires a careful consideration of how data filtering can influence community profiles. For instance, in samples with lower fungal assignment, this filtering can overrepresent the relative abundance of rare taxa, having effects on their real proportion and ecological significance within the community. Therefore, we encourage a cautious interpretation of the results, focusing on the potential of this data to understand broad fungal community patterns in *Puya.*

*Psilocybe* was the dominant genus detected in both roots and bulk soil. Members of this genus are commonly described as saprotrophs inhabiting soils, roots and other organic substrates, particularly in nitrogen-poor environments [59], such as páramo. Their high abundance in the endosphere fraction may reflect active colonization of senescing or decaying root tissue, or alternatively may be partly attributable to the limited resolution of the 18S V4–V5 region within Strophariaceae, a family that encompass multiple genera not easily distinguished by this marker and often requiring more variable sequences, such as the nLSU-rRNA [60]. We also detected a high relative abundance of putative pathotrophs in both sample types, including *Fusarium*, *Puccinia*, *Botrytis* and *Rhizoctonia*. Although *Bremia* infects aboveground tissues, related oomycetes can persist in soils through resistant oospores [61]. The ecological significance of these taxa in the context of *P. goudotiana* health, whether as active pathogens, opportunistic colonizers, or taxa whose pathogenic potential is context-dependent, cannot be resolved from amplicon data alone and warrants targeted investigation.

### Microbial functionality

An important caveat applies to the functional interpretations in this section. Bacterial functional profiles were predicted using FAPROTAX [43], which infers metabolic functions from genus-level taxonomic assignments based on cultivated relatives. This approach has known limitations: genera with few or no cultivated representatives (including several uncultured Acidobacteria and Xanthobacteraceae that were abundant in our samples) either receive no FAPROTAX annotation or are assigned functions extrapolated from distantly related cultivated taxa [62]. Similarly, fungal trophic modes assigned via FungalTraits represent primary lifestyle classifications derived from literature, which may not capture the functional versatility of individual taxa in a specific environmental context. The functional patterns described below should therefore be understood as ecologically plausible hypotheses grounded in taxonomic composition, rather than direct measurements of metabolic activity. Experimental validation through metagenomics, metatranscriptomics and enzymatic assays will be necessary to confirm these inferences.

With this in mind, several predicted bacterial functions were enriched in bulk soil, including fermentation, phototrophy, iron respiration, and functions associated with N and S cycling. Páramo soils are characterized by high organic matter accumulation resulting from low temperatures, high moisture, and slow decomposition rates [3]. These conditions may promote the formation of anoxic microsites associated with denitrification and sulfate reduction processes. The detection of predicted phototrophic and sulfur-related functions may also reflect microbial processes linked to exposed soil environments and organic matter turnover. Consistent with previous studies in páramo ecosystems, fermentation and phototrophic functions were particularly abundant, potentially associated with the higher abundance of *Rhodomicrobium* in bulk soil samples [63].

Root-associated communities showed higher abundance of *Jatrophihabitans* and *Rudaea.* These bacterial genera have previously been reported from plant tissues, soils and rhizosphere environments and include taxa associated with carbon metabolism and organic matter degradation [64–68]. Their increased abundance in roots may explain the greater representation of predicted chemoheterotrophic functions observed in root-associated communities, suggesting enrichment of taxa associated with decomposition and carbon utilization processes in the root endosphere. This predicted functional profile may enhance nutrient acquisition for *P. goudotiana* [69], supporting our second hypothesis of predicted functions related to organic matter utilization in the roots. Similar patterns have been reported in *Espeletia* spp., where pathways related to carbon degradation were more abundant in root-associated communities [26]. In addition, *Microbacterium* was also more abundant in the roots. Bacteria belonging to this genus are known to promote plant growth while activating plant defense pathways against pathogens [70,71]. Future studies should experimentally evaluate the antimicrobial activity of *Microbacterium* against root pathogens affecting *P. goudotiana*. Interestingly, nitrogen fixation was also more abundant in the roots. Although nitrogen-fixing bacteria have been isolated from the rhizosphere of other páramo plants like *Espeletia conglomerata* [72], this potential capability remains speculative for our system. Subsequent research utilizing transcriptomics or evaluating the expression of the nitrogenase reductase gene (*nif*H) is required to formally confirm the nitrogen-fixing activity of *Puya goudotiana* root-associated bacteria.

We also observed high abundance of saprotrophic fungi in both roots and bulk soil, with litter saprotrophs enriched in the roots. Saprotrophic fungi possess enzymatic capabilities associated with the decomposition of lignin and cellulose, contributing to organic matter turnover and nutrient mineralization [73]. Their presence in roots may reflect colonization of older root tissues enriched in complex structural compounds or broader decomposition processes occurring within the endosphere [74]. Alternatively, *P. goudotiana* may host saprotrophic fungi to enhance the breakdown of organic matter and mitigate the inhibitory effects of free aluminum on microbial activity commonly observed in páramo soils [75]. Because several saprotrophic ascomycetes can metabolize rhizodeposits [76], or the organic compounds released from the roots that include mucilage, exudates and sloughed cells/tissue, these fungi may also contribute to carbon cycling in root-associated environments. Further studies are needed to test these mechanisms and quantify the contribution of root-associated saprotrophs to plant nutrition.

Notably, and contrary to our second hypothesis, the arbuscular mycorrhizal lifestyle showed higher abundance in the bulk soil. This finding could be attributed to the limited taxonomic resolution of the 18S gene. *Rhizophagus* was the only mycorrhizal genus in our dataset. This genus has been previously found in higher abundance in the bulk soil compared to the rhizosphere, acting as a bridge between microbes from both compartments [77]. Furthermore, *Rhizophagus* has been described as one of the most common members of the family Glomeraceae in other terrestrial bromeliads [78]. As this is the first approach to arbuscular mycorrhizae that can potentially be associated with *Puya*, future studies focusing on these symbiotrophs should employ other molecular markers (SSU, ITS and/or LSU) to further detangle these associations.

### Network analysis

Contrary to our third hypothesis, bacterial, fungal and cross-domain co-occurrence networks exhibited higher complexity in the roots compared to bulk soil, despite the reduction in alpha diversity, especially within fungal communities. Although previous studies have suggested that the copiotroph-enriched root environment may constrain microbial interactions [12,79], our results indicate that root environments can simultaneously reduce diversity while promoting more structured co-occurrence patterns among dominant taxa. This pattern is consistent with rhizosphere filtering processes that favor recurrent associations among selected microbial groups [80].

Co-occurrence networks were dominated by positive correlations, suggesting that many taxa may share similar environmental preferences or exhibit potential ecological associations with the endosphere [81]. Increased network connectivity in roots may therefore reflect more structured microbial assemblages associated with root environments under high-altitude conditions. Especially in the cross-domain network, which may suggest potential cooperative relationships between fungi and bacteria that need to be further studied. Similar patterns have been reported in alpine forests, where higher root network complexity was associated with ectomycorrhizal fungal abundance [82]. In agricultural systems, root network complexity has also been linked to environmental variables such as water availability, pH and soil organic carbon [83], while geographic and regional factors may additionally influence network topology across spatial scales [84].

Although our results demonstrate the presence of highly connected root-associated networks, the relative contributions of environmental conditions, host filtering and microbial interactions to these patterns remain unresolved. Future studies integrating environmental variables, host traits and experimental approaches will be necessary to clarify the mechanisms underlying microbial network assembly in páramo ecosystems.

In addition, one limitation of this study is that DNA was extracted using different kits for root and bulk soil samples. Although both kits are commonly used for environmental microbiome studies, differences in extraction chemistry may influence DNA yield and the recovery of specific microbial taxa. Because DNA extraction kit was confounded with sample type, the relative contribution of biological differences and extraction-related bias cannot be discriminated. Consequently, differences between root and bulk soil communities should be interpreted with caution. Nonetheless, the clear separation observed in the ordination analyses, together with the PERMANOVA results, suggests that the differences are unlikely to be solely explained by extraction-related bias and could be aligned with the rhizosphere effect. Future studies should employ a single extraction protocol across all sample types to eliminate this potential source of bias.

## Conclusions

This study provides the first next-generation sequencing–based characterization of microbial communities associated with the genus *Puya.* The observed differences between root endosphere and bulk soil communities are consistent with rhizosphere filtering processes that selectively shape microbial assemblages near and within roots. Although the root endosphere niche exhibited lower taxonomic diversity than bulk soil, it supported a more complex and highly connected microbial co-occurrence network dominated by positive associations, a result contrary to our initial hypothesis and to findings from several other root-associated systems, which warrants further investigation. Exploratory 18S-based fungal profiling revealed broad community patterns that differed between compartments, though the lower taxonomic resolution of this marker relative to ITS means that fungal community structure should be interpreted cautiously and confirmed in future work using ITS amplicon sequencing.

Bulk soil functioned as a diverse microbial reservoir characterized by a higher prevalence of oligotrophic taxa and predicted functional groups associated with nutrient cycling. Because functional profiles were inferred from taxonomic data, further experimental studies integrating metagenomics, transcriptomics and enzymatic approaches will be necessary to determine the ecological roles of these microbial associations and their potential influence on plant nutrition and host performance under extreme high-altitude conditions. Overall, our findings establish a microbial baseline for future research on plant–microbe interactions in páramo ecosystems and provide information that may support conservation and restoration efforts involving *P. goudotiana* and other high-Andean plant species facing ongoing climate change.

## Supporting information

S1 Fig Study area and sampling site. (A) Location of the study area (green outline) within Colombia. (B) Extent of the Cruz Verde-Sumapaz páramo complex. (C) Detail of the Matarredonda Natural Reserve (outlined in brown) within the páramo complex. (D) Sampling site.
(TIF)

S2 Fig Principal Coordinates Analysis (PCoA) based on Bray–Curtis dissimilarities of microbial communities at the genus level. (A) Bacterial communities with the outlier. (B) Bacterial communities without the outlier. (C) Fungal communities with the outlier. (D) Fungal communities without the outlier.
(TIF)

S3 Fig Differentially abundant bacterial genera between sample types. Bars represent the log-fold change (LFC) derived from ANCOM-BC2 analysis, with error bars indicating the standard error (SE). Genera with positive LFC (purple) are significantly enriched in Root, while those with negative LFC (green) are significantly enriched in Bulk soil (*q* < 0.05, Holm correction).
(TIF)

S4 Fig Fungal co-occurrence networks of root and bulk soil samples. Node size is proportional to the degree number. Nodes are resolved at the genus level.
(TIF)

S1 Table Methods used to measure soil physicochemical properties in composite samples from each sampling zone. (PDF)

S2 Table Summary of soil physicochemical properties in the study area. (PDF)

S3 Table Relative abundance of the ten most abundant bacterial families according to sample type (Root or Bulk soil). (PDF)

S4 Table Relative abundance of the ten most abundant bacterial genera according to sample type (Root or Bulk soil). (PDF)

S5 Table Relative abundance of the ten most abundant fungal families according to sample type (Root or Bulk soil). (PDF)

S6 Table Relative abundance of the ten most abundant fungal genera according to sample type (Root or Bulk soil). (PDF)

S7 Table Significantly different bacterial predicted functions between sample types. Results from the Wilcoxon test include mean and standard deviation (sd) of relative abundances, test statistics (W) and *q-*values (FDR correction).
(PDF)

S8 Table Significantly different fungal trophic modes and lifestyles between sample types. Results from the Wilcoxon test include mean and standard deviation (sd) of relative abundances, test statistics (W) and *q-*values (FDR correction).
(PDF)

S9 Table Bacterial, fungal and cross-domain network topological metrics. (PDF)

## References

[1] Madriñán S, Cortés AJ, Richardson JE. Páramo is the world’s fastest evolving and coolest biodiversity hotspot. Front Genet. 2013;4:192. doi: 10.3389/fgene.2013.00192 24130570 PMC3793228

[2] Obando-Cabrera L, Díaz-Timoté JJ, Bastarrika A, Celis N, Hantson S. The Paramo Fire Atlas: quantifying burned area and trends across the tropical andes. Environ Res Lett. 2025;20(5):054019. doi: 10.1088/1748-9326/adc8ba

[3] Buytaert W, Célleri R, De Bièvre B, Cisneros F, Wyseure G, Deckers J, et al. Human impact on the hydrology of the Andean páramos. Earth Sci Rev. 2006;79(1–2):53–72. doi: 10.1016/j.earscirev.2006.06.002

[4] Padrón RS, Wilcox BP, Crespo P, Célleri R, Padrón RS, Wilcox BP. Rainfall in the Andean Páramo: New Insights from High-Resolution Monitoring in Southern Ecuador. J Hydrometeorol. 2015;16:985–96. doi: 10.1175/JHM-D-14-0135.1

[5] Morueta-Holme N, Engemann K, Sandoval-Acuña P, Jonas JD, Segnitz RM, Svenning J-C. Strong upslope shifts in Chimborazo’s vegetation over two centuries since Humboldt. Proc Natl Acad Sci U S A. 2015;112(41):12741–5. doi: 10.1073/pnas.1509938112 26371298 PMC4611603

[6] Sklenář P, Romoleroux K, Muriel P, Jaramillo R, Bernardi A, Diazgranados M, et al. Distribution changes in páramo plants from the equatorial high Andes in response to increasing temperature and humidity variation since 1880. Alp Bot. 2021;131(2):201–12. doi: 10.1007/s00035-021-00270-x

[7] Islam W, Noman A, Naveed H, Huang Z, Chen HYH. Role of environmental factors in shaping the soil microbiome. Environ Sci Pollut Res Int. 2020;27(33):41225–47. doi: 10.1007/s11356-020-10471-2 32829437

[8] Fierer N. Embracing the unknown: disentangling the complexities of the soil microbiome. Nat Rev Microbiol. 2017;15(10):579–90. doi: 10.1038/nrmicro.2017.87 28824177

[9] Vives-Peris V, de Ollas C, Gómez-Cadenas A, Pérez-Clemente RM. Root exudates: from plant to rhizosphere and beyond. Plant Cell Rep. 2020;39(1):3–17. doi: 10.1007/s00299-019-02447-5 31346716

[10] Compant S, Samad A, Faist H, Sessitsch A. A review on the plant microbiome: Ecology, functions, and emerging trends in microbial application. J Adv Res. 2019;19:29–37. doi: 10.1016/j.jare.2019.03.004 31341667 PMC6630030

[11] Bulgarelli D, Schlaeppi K, Spaepen S, Ver Loren van Themaat E, Schulze-Lefert P. Structure and functions of the bacterial microbiota of plants. Annu Rev Plant Biol. 2013;64:807–38. doi: 10.1146/annurev-arplant-050312-120106 23373698

[12] Ling N, Wang T, Kuzyakov Y. Rhizosphere bacteriome structure and functions. Nat Commun. 2022;13(1):836. doi: 10.1038/s41467-022-28448-9 35149704 PMC8837802

[13] Trivedi P, Leach JE, Tringe SG, Sa T, Singh BK. Plant-microbiome interactions: from community assembly to plant health. Nat Rev Microbiol. 2020;18(11):607–21. doi: 10.1038/s41579-020-0412-1 32788714

[14] Turner TR, James EK, Poole PS. The plant microbiome. Genome Biol. 2013;14(6):209. doi: 10.1186/gb-2013-14-6-209 23805896 PMC3706808

[15] Rajkumar M, Bruno LB, Banu JR. Alleviation of environmental stress in plants: The role of beneficial Pseudomonas spp. Crit Rev Environ Sci Technol. 2017;47:372–407. doi: 10.1080/10643389.2017.1318619

[16] Timofeeva AM, Galyamova MR, Sedykh SE. How do plant growth-promoting bacteria use plant hormones to regulate stress reactions?. Plants. 2024;13:2371. doi: 10.3390/plants13172371 39273855 PMC11397614

[17] Harrison MJ. Signaling in the arbuscular mycorrhizal symbiosis. Annu Rev Microbiol. 2005;59:19–42. doi: 10.1146/annurev.micro.58.030603.123749 16153162

[18] Hou M, Leng C, Zhu J, Yang M, Yin Y, Xing Y, et al. Alpine and subalpine plant microbiome mediated plants adapt to the cold environment: a systematic review. Environ Microbiome. 2024;19(1):82. doi: 10.1186/s40793-024-00614-0 39487507 PMC11529171

[19] Peyre G. What does the future hold for páramo plants? A modelling approach. Front Ecol Evol. 2022;10. doi: 10.3389/fevo.2022.896387

[20] Alzate-Guarín F, Murillo-Serna JS. Angiosperm flora on the páramos of northwestern Colombia: diversity and affinities. PhytoKeys. 2016;70:41–52. doi: 10.3897/phytokeys.70.8609 27829798 PMC5088703

[21] Hornung-Leoni CT. Bromeliads: traditional plant food in Latin America since prehispanic times. Polibotanica. 2011;32:219–29.

[22] Bernátková A, Pařiková A, Cisneros R, Čupić S, Ceacero F. Ecological effects on the nutritional value of bromeliads, and its influence on Andean bears’ diet selection. Ursus. 2021;2021(32e21). doi: 10.2192/ursus-d-20-00021.2

[23] Salinas L, Arana C, Suni M. El néctar de especies de Puya como recurso para picaflores Altoandinos de Ancash, Perú. Rev Peru Biol. 2007;14. doi: 10.15381/rpb.v14i1.2166

[24] Velásquez-Noriega P, Gómez-Díaz JA, Hornung-Leoni CT, Dáttilo W, Villalobos F, Krömer T. Unravelling the conservation status of the genus Puya (Bromeliaceae) across the Neotropics. Environ Monit Assess. 2025;197(12):1347. doi: 10.1007/s10661-025-14766-0 41247550

[25] Zizka A, Azevedo J, Leme E, Neves B, da Costa AF, Caceres D, et al. Biogeography and conservation status of the pineapple family (Bromeliaceae). Divers Distrib. 2020;26:183–95. doi: 10.1111/ddi.13004

[26] Ruiz-Pérez CA, Restrepo S, Zambrano MM. Microbial and Functional Diversity within the Phyllosphere of Espeletia Species in an Andean High-Mountain Ecosystem. Appl Environ Microbiol. 2016;82(6):1807–17. doi: 10.1128/AEM.02781-15 26746719 PMC4784022

[27] Lizarazo-Medina PX, Gómez-Vásquez D. Microbiota rizosférica de Espeletia spp. de los páramos de Santa inés y de Frontino-Urrao en Antioquia, Colombia. Acta Biolo Colomb. 2015;20:175–82. doi: 10.15446/abc.v20n1.42827

[28] Merino JM. Caracterización de microorganismos benéficos de montaña y de plantas obtenidos en tres zonas del Área Protegida Comunitaria Tambillo. Universidad Católica de Cuenca. 2025. https://dspace.ucacue.edu.ec/server/api/core/bitstreams/674d7395-55e2-44c5-ad64-de4235529b2b/content

[29] Palacios-Nazarit Y. Aislamiento de bacterias rizosféricas de Puya ochroleuca (Bromeliace) y su efecto sobre la germinación in vitro de semillas de Lycopersicum esculentum mill (tomate) como un modelo de interacción planta-microbio. Universidad de Caldas. 2024. https://repositorio.ucaldas.edu.co/handle/ucaldas/19792

[30] Cruz M, Lasso E. Insights into the functional ecology of páramo plants in Colombia. Biotropica. 2021;53(5):1415–31. doi: 10.1111/btp.12992

[31] Leon-Garcia IV, Lasso E. High heat tolerance in plants from the Andean highlands: Implications for paramos in a warmer world. PLoS One. 2019;14(11):e0224218. doi: 10.1371/journal.pone.0224218 31693675 PMC6834248

[32] Simmons T, Caddell DF, Deng S, Coleman-Derr D. Exploring the Root Microbiome: Extracting Bacterial Community Data from the Soil, Rhizosphere, and Root Endosphere. J Vis Exp. 2018;(135):57561. doi: 10.3791/57561 29782021 PMC6101100

[33] Lane DJ. 16S/23S rRNA sequencing. In: Stackebrandt E, Goodfellow M, editors. New York: John Wiley and Sons. 1991. p. 115–75.

[34] Hadziavdic K, Lekang K, Lanzen A, Jonassen I, Thompson EM, Troedsson C. Characterization of the 18S rRNA gene for designing universal eukaryote specific primers. PLoS One. 2014;9(2):e87624. doi: 10.1371/journal.pone.0087624 24516555 PMC3917833

[35] De Coster W, D’Hert S, Schultz DT, Cruts M, Van Broeckhoven C. NanoPack: visualizing and processing long-read sequencing data. Bioinformatics. 2018;34(15):2666–9. doi: 10.1093/bioinformatics/bty149 29547981 PMC6061794

[36] Wood DE, Lu J, Langmead B. Improved metagenomic analysis with Kraken 2. Genome Biol. 2019;20(1):257. doi: 10.1186/s13059-019-1891-0 31779668 PMC6883579

[37] Quast C, Pruesse E, Yilmaz P, Gerken J, Schweer T, Yarza P, et al. The SILVA ribosomal RNA gene database project: improved data processing and web-based tools. Nucleic Acids Res. 2013;41(D1):D590-6. doi: 10.1093/nar/gks1219 23193283 PMC3531112

[38] Breitwieser FP, Salzberg SL. Pavian: interactive analysis of metagenomics data for microbiome studies and pathogen identification. Bioinformatics. 2020;36(4):1303–4. doi: 10.1093/bioinformatics/btz715 31553437 PMC8215911

[39] R Core Team. R: A Language and Environment for Statistical Computing. 2025.

[40] McMurdie PJ, Holmes S. phyloseq: an R package for reproducible interactive analysis and graphics of microbiome census data. PLoS One. 2013;8(4):e61217. doi: 10.1371/journal.pone.0061217 23630581 PMC3632530

[41] Lin H, Peddada SD. Multigroup analysis of compositions of microbiomes with covariate adjustments and repeated measures. Nat Methods. 2024;21(1):83–91. doi: 10.1038/s41592-023-02092-7 38158428 PMC10776411

[42] Oksanen J, Simpson GL, Blanchet F, Kindt R, Legendre P, Minchin P. vegan Community Ecology Package. 2025. doi: 10.32614/CRAN.package.vegan

[43] Louca S, Parfrey LW, Doebeli M, New Collective Author. Decoupling function and taxonomy in the global ocean microbiome. Science. 2016;353(6305):1272–7. doi: 10.1126/science.aaf4507 27634532

[44] Põlme S, Abarenkov K, Nilsson RH, Lindahl BD, Clemmensen KE, Kauserud H, et al. FungalTraits: a user-friendly traits database of fungi and fungus-like stramenopiles. Fungal Divers. 2020;105:1–16. doi: 10.1007/s13225-020-00466-2

[45] Salamanca-Fonseca M, Sanchez A, Corrales A, Kauserud H, Thoen E, Krabberød AK, et al. Beyond seasonal and host factors: ecosystem dynamics drive palm-associated root fungal communities at a local scale. Plant Soil. 2025;514(1):1027–41. doi: 10.1007/s11104-025-07438-y

[46] Liu C, Cui Y, Li X, Yao M. microeco: an R package for data mining in microbial community ecology. FEMS Microbiol Ecol. 2021;97(2):fiaa255. doi: 10.1093/femsec/fiaa255 33332530

[47] Liu C, Li C, Jiang Y, Zeng RJ, Yao M, Li X. A guide for comparing microbial co-occurrence networks. Imeta. 2023;2(1):e71. doi: 10.1002/imt2.71 38868345 PMC10989802

[48] Banerjee S, Schlaeppi K, van der Heijden MGA. Keystone taxa as drivers of microbiome structure and functioning. Nat Rev Microbiol. 2018;16(9):567–76. doi: 10.1038/s41579-018-0024-1 29789680

[49] Yon GGV. Building, importing, and exporting GEXF graph files with rgexf. J Open Source Softw. 2021;6:3456. doi: 10.21105/joss.03456

[50] Bastian M, Heymann S, Jacomy M. Gephi: An open source software for exploring and manipulating networks visualization and exploration of large graphs. In: Proceedings of the International AAAI Conference on Web and Social Media. 2009. doi: 10.13140/2.1.1341.1520

[51] Fruchterman TMJ, Reingold EM. Graph drawing by force‐directed placement. Softw Pract Exp. 1991;21:1129–64. doi: 10.1002/spe.4380211102

[52] Dragone NB, Hoffert M, Strickland MS, Fierer N. Taxonomic and genomic attributes of oligotrophic soil bacteria. ISME Commun. 2024;4(1):ycae081. doi: 10.1093/ismeco/ycae081 38988701 PMC11234899

[53] Llambí LD, Soto A, Célleri R, De Bievre B, Ochoa B, Borja P. Ecología, hidrología y suelos de páramos. 1st ed. Proyecto Páramo Andino. 2012.

[54] Reyes-Ardila WL, Vélez-Martínez GA, Duque-Zapata JD, Rugeles-Silva PA, Muñoz Flórez JE, López-Álvarez D. Exploring soil bacterial and fungal communities in Colombian terrestrial ecosystems modulated by altitude-influenced factors. PLoS One. 2024;19(12):e0312842. doi: 10.1371/journal.pone.0312842 39666620 PMC11637269

[55] Pankratov TA, Dedysh SN. Granulicella paludicola gen. nov., sp. nov., Granulicella pectinivorans sp. nov., Granulicella aggregans sp. nov. and Granulicella rosea sp. nov., acidophilic, polymer-degrading acidobacteria from Sphagnum peat bogs. Int J Syst Evol Microbiol. 2010;60:2951–9. doi: 10.1099/ijs.0.021824-0 20118293

[56] Okamura K, Kawai A, Yamada T, Hiraishi A. Acidipila rosea gen. nov., sp. nov., an acidophilic chemoorganotrophic bacterium belonging to the phylum Acidobacteria. FEMS Microbiol Lett. 2011;317:138–42. doi: 10.1111/j.1574-6968.2011.02224.x 21255071

[57] Kulichevskaya IS, Danilova OV, Tereshina VM, Kevbrin VV, Dedysh SN. Descriptions of Roseiarcus fermentans gen. nov., sp. nov., a bacteriochlorophyll a-containing fermentative bacterium related phylogenetically to alphaproteobacterial methanotrophs, and of the family Roseiarcaceae fam. nov. Int J Syst Evol Microbiol. 2014;64:2558–65. doi: 10.1099/ijs.0.064576-0 24812364

[58] Zafra G, Rangel Ibañez D. Diversidad microbiana asociada a Espeletia spp. en ecosistemas de alta montaña. Biotecnología en el Sector Agropecuario y Agroindustrial. 2022;20:129–41. doi: 10.18684/rbsaa.v20.n2.2022.1640

[59] Meyer M, Slot J. The evolution and ecology of psilocybin in nature. Fungal Genet Biol. 2023;167:103812. doi: 10.1016/j.fgb.2023.103812 37210028

[60] Moncalvo JM, Lutzoni FM, Rehner SA, Johnson J, Vilgalys R. Phylogenetic relationships of agaric fungi based on nuclear large subunit ribosomal DNA sequences. Syst Biol. 2000;49:278–305. doi: 10.1093/sysbio/49.2.278 12118409

[61] Drenth A, Janssen EM, Govers F. Formation and survival of oospores of Phytophthora infestans under natural conditions. Plant Pathol. 1995;44(1):86–94. doi: 10.1111/j.1365-3059.1995.tb02719.x

[62] Sansupa C, Wahdan SFM, Hossen S, Disayathanoowat T, Wubet T, Purahong W. Can we use functional annotation of prokaryotic taxa (faprotax) to assign the ecological functions of Soil Bacteria?. Appl Sci. 2021;11(2):688. doi: 10.3390/app11020688

[63] Vélez-Martínez GA, Reyes-Ardila WL, Duque-Zapata JD, Rugeles-Silva PA, Muñoz Flórez JE, López-Álvarez D. Soil bacteria and fungi communities are shaped by elevation influences in Colombian forest and páramo natural ecosystems. Int Microbiol. 2024;27(2):377–91. doi: 10.1007/s10123-023-00392-8 37458953 PMC10991037

[64] Guo L, Zheng S, Cao C, Li C. Tillage practices and straw-returning methods affect topsoil bacterial community and organic C under a rice-wheat cropping system in central China. Sci Rep. 2016;6:33155. doi: 10.1038/srep33155 27611023 PMC5017303

[65] Suh MK, Kim J-S, Eom MK, Kim HS, Do HE, Shin YK, et al. Jatrophihabitans cynanchi sp. nov., isolated from rhizosphere soil of Cynanchum wilfordii. Antonie Van Leeuwenhoek. 2024;117(1):19. doi: 10.1007/s10482-023-01902-4 38189847

[66] Madhaiyan M, Hu CJ, Kim SJ, Weon HY, Kwon SW, Ji L. Jatrophihabitans endophyticus gen. nov., sp. nov., an endophytic actinobacterium isolated from a surface-sterilized stem of Jatropha curcas L. Int J Syst Evol Microbiol. 2013;63:1241–8. doi: 10.1099/ijs.0.039685-0 22798659

[67] Zhu G, Schmidt O, Luan L, Xue J, Fan J, Geisen S, et al. Bacterial Keystone Taxa Regulate Carbon Metabolism in the Earthworm Gut. Microbiol Spectr. 2022;10(5):e0108122. doi: 10.1128/spectrum.01081-22 35972247 PMC9603485

[68] Kim S-J, Moon J-Y, Lim J-M, Hamada M, Ahn J-H, Weon H-Y, et al. Jatrophihabitans soli sp. nov., isolated from soil. Int J Syst Evol Microbiol. 2015;65(Pt 6):1759–63. doi: 10.1099/ijs.0.000173 25744581

[69] Kasozi N, Kaiser H, Wilhelmi B. Metabarcoding Analysis of Bacterial Communities Associated with Media Grow Bed Zones in an Aquaponic System. Int J Microbiol. 2020;2020:8884070. doi: 10.1155/2020/8884070 33061984 PMC7547338

[70] Berendsen RL, Vismans G, Yu K, Song Y, de Jonge R, Burgman WP, et al. Disease-induced assemblage of a plant-beneficial bacterial consortium. ISME J. 2018;12(6):1496–507. doi: 10.1038/s41396-018-0093-1 29520025 PMC5956071

[71] Patel A, Sahu KP, Mehta S, Javed M, Balamurugan A, Ashajyothi M, et al. New Insights on Endophytic Microbacterium-Assisted Blast Disease Suppression and Growth Promotion in Rice: Revelation by Polyphasic Functional Characterization and Transcriptomics. Microorganisms. 2023;11(2):362. doi: 10.3390/microorganisms11020362 36838327 PMC9963279

[72] Carreño León JV, Fuentes-Marín S, Ibañez DR, Fajardo-López M, Archila-Durán L, Zafra G. Culturable Plant Growth-Promoting Bacteria from a High-Altitude Páramo Soil Ecosystem as Potential Sources of Biofertilizers. Revista de la Universidad Industrial de Santander Salud. 2026;58. doi: 10.18273/saluduis.58.e26v58a02

[73] Crowther TW, Boddy L, Hefin Jones T. Functional and ecological consequences of saprotrophic fungus-grazer interactions. ISME J. 2012;6(11):1992–2001. doi: 10.1038/ismej.2012.53 22717883 PMC3475375

[74] de Boer W, Kowalchuk GA, van Veen JA. “Root-food” and the rhizosphere microbial community composition. New Phytol. 2006;170(1):3–6. doi: 10.1111/j.1469-8137.2006.01674.x 16539597

[75] Bottner P, Pansu M, Sarmiento L, Hervé D, Callisaya-Bautista R, Metselaar K. Factors controlling decomposition of soil organic matter in fallow systems of the high tropical Andes: A field simulation approach using 14C- and 15N-labelled plant material. Soil Biology and Biochemistry. 2006;38(8):2162–77. doi: 10.1016/j.soilbio.2006.01.029

[76] Hannula SE, Boschker HTS, de Boer W, van Veen JA. 13C pulse-labeling assessment of the community structure of active fungi in the rhizosphere of a genetically starch-modified potato (Solanum tuberosum) cultivar and its parental isoline. New Phytol. 2012;194(3):784–99. doi: 10.1111/j.1469-8137.2012.04089.x 22413848

[77] Shami A, Jalal RS, Ashy RA, Abuauf HW, Baz L, Refai MY, et al. Use of Metagenomic Whole Genome Shotgun Sequencing Data in Taxonomic Assignment of Dipterygium glaucum Rhizosphere and Surrounding Bulk Soil Microbiomes, and Their Response to Watering. Sustainability. 2022;14(14):8764. doi: 10.3390/su14148764

[78] Leroy C, Maes AQ, Louisanna E, Schimann H, Séjalon-Delmas N. Taxonomic, phylogenetic and functional diversity of root-associated fungi in bromeliads: effects of host identity, life forms and nutritional modes. New Phytol. 2021;231(3):1195–209. doi: 10.1111/nph.17288 33605460

[79] Wei X, Fu T, He G, Zhong Z, Yang M, Lou F, et al. Characteristics of rhizosphere and bulk soil microbial community of Chinese cabbage (Brassica campestris) grown in Karst area. Front Microbiol. 2023;14:1241436. doi: 10.3389/fmicb.2023.1241436 37789857 PMC10542900

[80] Shi S, Nuccio EE, Shi ZJ, He Z, Zhou J, Firestone MK. The interconnected rhizosphere: High network complexity dominates rhizosphere assemblages. Ecol Lett. 2016;19(8):926–36. doi: 10.1111/ele.12630 27264635

[81] Faust K, Raes J. Microbial interactions: from networks to models. Nat Rev Microbiol. 2012;10(8):538–50. doi: 10.1038/nrmicro2832 22796884

[82] Wang D, Deng S, Yang H, Li N, Feng Q, Liu J, et al. The microbial network exhibits higher complexity in the rhizosphere than in bulk soils along elevational gradients in the alpine forests. Appl Soil Ecol. 2025;213:106264. doi: 10.1016/j.apsoil.2025.106264

[83] Guo Y, Kuzyakov Y, Li N, Song B, Liu Z, Adams JM, et al. Rice rhizosphere microbiome is more diverse but less variable along environmental gradients compared to bulk soil. Plant Soil. 2024;506:767–85. doi: 10.1007/s11104-024-06728-1

[84] Zhang B, Zhang J, Liu Y, Shi P, Wei G. Co-occurrence patterns of soybean rhizosphere microbiome at a continental scale. Soil Biol Biochem. 2018;118:178–86. doi: 10.1016/j.soilbio.2017.12.011

