# Supplementary material for: Root-associated bacterial and fungal communities of the endangered páramo bromeliad Puya goudotiana
Source: PLoS One. 2026 Aug 31;21(8):e0357104. doi: 10.1371/journal.pone.0357104 (PMC13529022; doi:10.1371/journal.pone.0357104)
Supplement: Supplementary file 1 [file pone.0357104.s005.pdf]

**Soil property**	**Method**
pH	Potentiometric in relation to soil/water 1:1
Exchangeable bases (Ca, Mg, P and Na)	Extraction with ammonium acetate (1M, pH 7) and quantification by atomic absorption-emission
Organic carbon	Walkey-Black Method
Soil texture	Bouyoucos Method (hydrometer) adapted for Colombian soils
Cation exchange capacity	Extraction with ammonium acetate (1M, pH 7) and volumetric quantification

All analyses were conducted with temperatures between 10-14°C and relative humidity not exceeding 85%
