# Supplementary material for: Root-associated bacterial and fungal communities of the endangered páramo bromeliad Puya goudotiana
Source: PLoS One. 2026 Aug 31;21(8):e0357104. doi: 10.1371/journal.pone.0357104 (PMC13529022; doi:10.1371/journal.pone.0357104)
Supplement: Supplementary file 2 [file pone.0357104.s006.pdf]

**Soil variable**	**n**	**Mean±sd**
pH	8	4.58±0.49
Total Nitrogen (%)	8	1.31±0.39
Effective Cation Exchange Capacity (ECEC) (meq/100g)	7	6.34±4.88
Oxidizable Organic Carbon (OOC) (%)	8	13.55±2.48
Organic Matter (%)	8	44.56±19.50
Clay (%)	8	9.71±3.16
Sand (%)	8	42.85±28.90
Silt (%)	8	47.44±26.36
K (mg/kg)	8	134.92±28.20
Ca (mg/kg)	8	38.31±41.82
Mg (mg/kg)	8	44.56±19.50
Na (mg/kg)	8	16.73±6.11
P (mg/kg)	8	20.74±25.29
Ca/K	8	0.52±0.46
Ca/Mg	8	0.45±0.26
Mg/K	8	1.06±0.34
(Ca+Mg)/K	8	1.58±0.75

n=8 for all parameters except for ECEC (n=7), as this value was not provided by the external laboratory
