# Supplementary material for: Root-associated bacterial and fungal communities of the endangered páramo bromeliad Puya goudotiana
Source: PLoS One. 2026 Aug 31;21(8):e0357104. doi: 10.1371/journal.pone.0357104 (PMC13529022; doi:10.1371/journal.pone.0357104)
Supplement: Supplementary file 3 [file pone.0357104.s007.pdf]

**Type**	**Phylum**	**Family**	**Relative abundance (%)**
Root	Acidobacteria	Acidobacteriaceae (Subgroup 1)	13.40
	Proteobacteria	Xanthobacteraceae	13.10
	Proteobacteria	Beijerinckiaceae	8.00
	Moraxellaceae	Moraxellaceae	5.52
	Planctomycetes	Isosphaeraceae	3.69
	Proteobacteria	Burkholderiaceae	3.54
	Proteobacteria	Unknown family Gammaproteobacteria	3.12
	Proteobacteria	Acetobacteraceae	3.05
	Actinobacteria	Acidothermaceae	2.76
	Proteobacteria	Diplorickettsiaceae	2.65
Bulk soil	Acidobacteria	Uncultured (Acidobacteriales)	11.90
	Planctomycetes	Gemmataceae	9.29
	Acidobacteria	Solibacteraceae (Subgroup 3)	8.63
	Proteobacteria	Xanthobacteraceae	7.09
	Chloroflexi	Ktedonobacteraceae	6.20
	Verrucomicrobia	Pedosphaeraceae	5.46
	Proteobacteria	Beijerinckiaceae	4.61
	Acidobacteria	Acidobacteriaceae (Subgroup 1)	3.56
	Acidobacteria	Koribacteraceae	3.40
	Planctomycetes	Isosphaeraceae	2.46
