# Supplementary material for: Root-associated bacterial and fungal communities of the endangered páramo bromeliad Puya goudotiana
Source: PLoS One. 2026 Aug 31;21(8):e0357104. doi: 10.1371/journal.pone.0357104 (PMC13529022; doi:10.1371/journal.pone.0357104)
Supplement: Supplementary file 4 [file pone.0357104.s008.pdf]

Type	Phylum	Family	Genus	Relative abundance (%)
Root	Acidobacteria	Acidobacteriaceae	*Granulicella*	7.81
	Proteobacteria	Xanthobacteraceae	*Bradyrhizobium*	7.32
	Proteobacteria	Beijerinckiaceae	*Roseiarcus*	6.49
	Proteobacteria	Moraxellaceae	*Acinetobacter*	5.54
	Proteobacteria	Xanthobacteraceae	Uncultured (Xanthobacteraceae)	4.74
	Acidobacteria	Acidobacteriaceae	*Acidipila*	4.49
	Actinobacteria	Acidothermaceae	*Acidothermus*	3.19
	Proteobacteria	Unknown family	*Acidibacter*	2.95
	Proteobacteria	Burkholderiaceae	*Burkholderia-Caballeronia-Paraburkholderia*	2.45
	Planctomycetes	Isosphaeraceae	*Singulisphaera*	2.09
Bulk soil	Planctomycetes	Gemmataceae	Uncultured (Gemmataceae)	12.30
	Planctomycetes	Gemmataceae	*Candidatus Solibacter*	6.38
	Proteobacteria	Xanthobacteraceae	Uncultured (Xanthobacteraceae)	5.91
	Proteobacteria	Beijerinckiaceae	*Roseiarcus*	5.20
	Chloroflexi	Ktedonobacteraceae	*HSB OF53-F07*	5.20
	Acidobacteria	Solibacteraceae	*Bryobacter*	5.03
	Acidobacteria	Koribacteraceae	*Candidatus Koribacter*	4.64
	Verrucomicrobia	Pedosphaeraceae	*ADurb.Bin063-1*	3.00
	Proteobacteria	Rhodomicrobiaceae	*Rhodomicrobium*	2.25
	Verrucomicrobia	Xiphinematobacteraceae	*Candidatus Xiphinematobacter*	2.06
