# Supplementary material for: Root-associated bacterial and fungal communities of the endangered páramo bromeliad Puya goudotiana
Source: PLoS One. 2026 Aug 31;21(8):e0357104. doi: 10.1371/journal.pone.0357104 (PMC13529022; doi:10.1371/journal.pone.0357104)
Supplement: Supplementary file 5 [file pone.0357104.s009.pdf]

Type	Phylum	Family	Relative abundance (%)
Root	Basidiomycota	Strophariaceae	33.10
	Ascomycota	Nectriaceae	11.30
	Basidiomycota	Ceratobasidiaceae	8.18
	Ascomycota	Sclerotiniaceae	7.78
	Basidiomycota	Marasmiaceae	6.84
	Basidiomycota	Pucciniaceae	6.55
	Ascomycota	Mycosphaerellaceae	2.56
	Ascomycota	Aspergillaceae	2.47
	Basidiomycota	Trichosporonaceae	2.30
	Ascomycota	Sordariaceae	2.30
Bulk soil	Ascomycota	Podosporaceae	11.50
	Ascomycota	Nectriaceae	10.40
	Basidiomycota	Strophariaceae	8.71
	Ascomycota	Sclerotiniaceae	8.21
	Basidiomycota	Cryptococcaceae	7.01
	Basidiomycota	Pucciniaceae	6.82
	Ascomycota	Sordariaceae	5.95
	Glomeromycota	Glomeraceae	4.73
	Ascomycota	Pleosporaceae	4.70
	Basidiomycota	Ceratobasidiaceae	4.07
