# Supplementary material for: Root-associated bacterial and fungal communities of the endangered páramo bromeliad Puya goudotiana
Source: PLoS One. 2026 Aug 31;21(8):e0357104. doi: 10.1371/journal.pone.0357104 (PMC13529022; doi:10.1371/journal.pone.0357104)
Supplement: Supplementary file 6 [file pone.0357104.s010.pdf]

Type	Phylum	Family	Genus	Relative abundance (%)
Root	Basidiomycota	Strophariaceae	*Psilocybe*	27.20
	Ascomycota	Nectriaceae	*Fusarium*	13.70
	Basidiomycota	Ceratobasidiaceae	*Rhizoctonia*	10.20
	Ascomycota	Sclerotiniaceae	*Botrytis*	8.93
	Basidiomycota	Pucciniaceae	*Puccinia*	7.30
	Basidiomycota	Marasmiaceae	*Marasmius*	7.25
	Oomycota	Peronosporaceae	*Bremia*	2.54
	Basidiomycota	Trichosporonaceae	*Vanrija*	2.31
	Ascomycota	Didymellaceae	*Ascochyta*	2.07
	Ascomycota	Trichomonascaceae	*Sugiyamaella*	2.05
Bulk soil	Ascomycota	Podosporaceae	*Podospora*	11.80
	Ascomycota	Nectriaceae	*Fusarium*	10.80
	Basidiomycota	Strophariaceae	*Psilocybe*	8.77
	Ascomycota	Sclerotiniaceae	*Botrytis*	8.49
	Basidiomycota	Pucciniaceae	*Puccinia*	7.02
	Basidiomycota	Cryptococcaceae	*Kwoniella*	6.83
	Ascomycota	Sordariaceae	*Neurospora*	5.02
	Glomeromycota	Glomeraceae	*Rhizophagus*	4.80
	Oomycota	Peronosporaceae	*Bremia*	4.18
	Ascomycota	Pleosporaceae	*Pyrenophora*	4.10
