# Supplementary material for: Root-associated bacterial and fungal communities of the endangered páramo bromeliad Puya goudotiana
Source: PLoS One. 2026 Aug 31;21(8):e0357104. doi: 10.1371/journal.pone.0357104 (PMC13529022; doi:10.1371/journal.pone.0357104)
Supplement: Supplementary file 7 [file pone.0357104.s011.pdf]

**Function**	**Root (Mean±sd)**	**Bulk soil (Mean±sd)**	**W**	***q*-value**
aerobic ammonia oxidation	0.001±0.0014	0.0213±0.03	127	0.00270
aerobic chemoheterotrophy	0.3417±0.0377	0.2119±0.0397	1	0.00009
animal parasites or symbionts	0.0126±0.0069	0.0595±0.0483	130	0.00146
anoxygenic photoautotrophy	0.0014±9e-04	0.0037±9e-04	139	0.00013
anoxygenic photoautotrophy S oxidizing	0.0014±9e-04	0.0037±9e-04	138	0.00014
chemoheterotrophy	0.3544±0.035	0.2579±0.0339	2	0.00009
chlorate reducers	0	1e-04±2e-04	122	0.00483
dark hydrogen oxidation	2e-04±2e-04	5e-04±5e-04	118	0.01926
dark iron oxidation	3e-04±6e-04	0.0011±0.0028	110	0.04789
denitrification	0.0014±9e-04	0.0039±0.0011	139	0.00013
fermentation	0.0065±0.0094	0.0304±0.0119	132	0.00091
human associated	0.0095±0.0045	0.0052±0.0022	22	0.00734
human pathogens all	0.0091±0.0045	0.0048±0.0022	23	0.00881
intracellular parasites	0.0069±0.0045	0.0183±0.0098	120	0.01053
iron respiration	2e-04±2e-04	0.008±0.0072	143	0.00024
nitrate denitrification	0.0014±9e-04	0.0039±0.0011	139	0.00013
nitrate reduction	0.0092±0.0042	0.024±0.0129	125	0.00392
nitrification	0.0026±0.0019	0.0248±0.0297	126	0.00319
nitrite denitrification	0.0014±9e-04	0.0039±0.0011	139	0.00013
nitrite respiration	0.0014±9e-04	0.004±0.0011	139	0.00013
nitrogen fixation	0.0749±0.0315	0.0428±0.0211	28	0.02182
oxygenic photoautotrophy	2e-04±5e-04	0.0014±0.0017	131	0.00243
photoautotrophy	0.0016±0.0013	0.0051±0.0016	137	0.00019
photoheterotrophy	0.0105±0.0118	0.0621±0.0431	138	0.00014
photosynthetic cyanobacteria	2e-04±5e-04	0.0014±0.0017	131	0.00243
phototrophy	0.0108±0.0119	0.0635±0.0426	138	0.00014
predatory or exoparasitic	0.0058±0.0041	0.0248±0.0183	127	0.00270
respiration of sulfur compounds	7e-04±0.0015	0.0047±0.0041	136	0.00024
sulfate respiration	6e-04±0.0015	0.0046±0.0041	136	0.00103
thiosulfate respiration	0	1e-04±2e-04	120	0.00319
