# Supplementary material for: Root-associated bacterial and fungal communities of the endangered páramo bromeliad Puya goudotiana
Source: PLoS One. 2026 Aug 31;21(8):e0357104. doi: 10.1371/journal.pone.0357104 (PMC13529022; doi:10.1371/journal.pone.0357104)
Supplement: Supplementary file 8 [file pone.0357104.s012.pdf]

| **Trophic mode*/fungal lifestyle** | **Root (Mean±sd)** | **Bulk soil (Mean±sd)** | **W** | ***q*-value** |
|---|---|---|---|---|
| symbiotroph* | 0.0123±0.0168 | 0.0479±0.0304 | 127 | 0.00343 |
| dung saprotroph | 0.0278±0.0137 | 0.1726±0.1556 | 144 | 0.00001 |
| nectar/tap saprotroph | 0.0045±0.0032 | 0.0242±0.0088 | 142 | 0.00002 |
| unspecified saprotroph | 0.0429±0.0304 | 0.1108±0.0360 | 136 | 0.00018 |
| arbuscular mycorrhizal | 0.0124±0.0168 | 0.0480±0.0304 | 127 | 0.00236 |
| soil saprotroph | 0.0144±0.0144 | 0.0339±0.0151 | 122 | 0.00641 |
| litter saprotroph | 0.3449±0.2377 | 0.1239±0.0935 | 24 | 0.00827 |
