# Supplementary material for: Root-associated bacterial and fungal communities of the endangered páramo bromeliad Puya goudotiana
Source: PLoS One. 2026 Aug 31;21(8):e0357104. doi: 10.1371/journal.pone.0357104 (PMC13529022; doi:10.1371/journal.pone.0357104)
Supplement: Supplementary file 9 [file pone.0357104.s013.pdf]

Bacteria		
**Metric**	**Root**	**Bulk soil**
Vertex	143	25
Edge	842	16
Average degree	11.776	1.28
Average path length	2.725	1.639
Network diameter	9	4
Clustering coefficient	0.547	0.273
Density	0.083	0.053
Heterogeneity	0.998	0.576
Centralization	0.192	0.113
Modularity	0.320	0.774
**Fungi**		
**Metric**	**Root**	**Bulk soil**
Vertex	26	3
Edge	48	2
Average degree	3.692	1.333
Average path length	2.008	1.236
Network diameter	4	2
Clustering coefficient	0.446	0
Density	0.148	0.667
Heterogeneity	0.738	0.433
Centralization	0.252	0.333
Modularity	0.445	1.80 e-16
**Cross-domain (Bacteria-Fungi)**		
**Metric**	**Root**	**Bulk soil**
Vertex	143	22
Edge	654	12
Average degree	9.147	1.091
Average path length	3.578	1.069
Network diameter	12	2
Clustering coefficient	0.529	0
Density	0.064	0.052
Heterogeneity	1.007	0.269
Centralization	0.182	0.043
Modularity	0.401	0.889
